# Mitophagy Improves Ethanol Tolerance in Yeast: Regulation by Mitochondrial Reactive Oxygen Species in *Saccharomyces cerevisiae*

**DOI:** 10.4014/jmb.2004.04073

**Published:** 2020-09-25

**Authors:** Hongjuan Jing, Huanhuan Liu, Zhang Lu, Cui liuqing,, Xiaorong Tan

**Affiliations:** College of Biological Engineering, Henan University of Technology, Zhengzhou 450001, P.R. China

**Keywords:** Ethanol stress, mitophagy, reactive oxygen species, hydrogen peroxide, superoxide anion

## Abstract

Ethanol often accumulates during the process of wine fermentation, and mitophagy has critical role in ethanol output. However, the relationship between mitophagy and ethanol stress is still unclear. In this study, the expression of *ATG11* and *ATG32* genes exposed to ethanol stress was accessed by real-time quantitative reverse transcription polymerase chain reaction (qRT-PCR). The result indicated that ethanol stress induced expression of the *ATG11* and *ATG32* genes. The colony sizes and the alcohol yield of *atg11* and *atg32* were also smaller and lower than those of wild type strain under ethanol whereas the mortality of mutants is higher. Furthermore, compared with wild type, the membrane integrity and the mitochondrial membrane potential of *atg11* and *atg32* exhibited greater damage following ethanol stress. In addition, a greater proportion of mutant cells were arrested at the G1/G0 cell cycle. There was more aggregation of peroxide hydrogen (H_2_O_2_) and superoxide anion (O_2_^•-^) in mutants. These changes in H_2_O_2_ and O_2_^•-^ in yeasts were altered by reductants or inhibitors of scavenging enzyme by means of regulating the expression of *ATG11* and *ATG32* genes. Inhibitors of the mitochondrial electron transport chain (mtETC) also increased production of H_2_O_2_ and O_2_^•-^ by enhancing expression of the *ATG11* and *ATG32* genes. Further results showed that activator or inhibitor of autophagy also activated or inhibited mitophagy by altering production of H_2_O_2_ and O_2_^•^. Therefore, ethanol stress induces mitophagy which improves yeast the tolerance to ethanol and the level of mitophagy during ethanol stress is regulated by ROS derived from mtETC.

## Introduction

In eukaryotes, mitochondria are the main site of reactive oxygen species (ROSs) production. For example, superoxide anion (O_2_^•-^) is produced by complex I and complex III of the electron transport chain in mitochondria (mtETC) [1, 2]. Subsequently, O_2_^•-^ is immediately converted into less-reactive hydrogen peroxide (H_2_O_2_) by mitochondrial manganese superoxide dismutase (Mn-SOD) [3-5]. Catalase (CAT) in mitochondria then converts H_2_O_2_ into water [6-8]. Therefore, the inhibition of Mn-SOD and CAT leads to increase of ROS in both cytoplasm and mitochondria. Mitochondria are initially injured by ROS, then the injured mitochondria produce more ROS, initiating a progressive cycle of damage. Therefore, preventing mitochondrial dysfunction is especially important for the maintenance of cellular homeostasis [9, 10]. Several studies suggest that mitophagy has a vital role in mitochondrial quality control by eliminating redundant, injured and disfunctional mitochondria [9-13].

Mitophagy is a form of selective macroautophagy (hereafter referred to as autophagy) characterized by specific degradation mitochondria. A series of autophagy related genes (ATG)s have been identified in the budding yeast *Saccharomyces cerevisiae* [14, 15]. Besides common autophagy related proteins, the completion of mitophagy requires additional specific proteins. A mitochondrial outer membrane protein *ATG32*, which function as a receptor for mitophagy, plays a key role in the regulation of mitophagy. The protein *ATG32* recruits ATG8 and *ATG11* to the surface of mitochondria to form a mitophagy initiation complex [10, 12, 16]. The inhibition expression of *ATG32* leads to the suppression of mitophagy, and vice versa. The mutant *atg 32* inhibits mitophagy, but has no effect on common autophagy [10]. In contrast, a key determinant for mitophagy efficiency is the induction of expression of the *ATG32* gene [17]. Yeast *ATG11*, as the structural scaffold, participates in mitophagy by mean of interaction with receptor *ATG32* [10,12,18,19]. Except that mitophagy, *ATG11* also participates in other forms of selective autophagy [19], such as pexophage [20], ER-phagy [21], genotoxin-induced targeted autophagy (Eapena *et al*., 2017) [22], and ribophagy [23].

There is evidence that ROS production is elevated during the fermentation process [24], and that the production of ROS might also be a component of the yeast cellular response to a variety of fermentation stressors [25, 26]. Zyrina *et al*. has reported that H_2_O_2_ in the mitochondrial matrix has a signaling role in ethanol tolerance by Yap1p [5]. Additional studies indicate that mild ROS, as a signal molecule, regulates mitophagy [10-12,27].

With regards to improving ethanol output, it has been reported that *S. cerevisiae* disrupts mitophagy function [28]. Our group has proved that ethanol induces autophagy [29]. However, there are no published studies on the relationship between mitophagy and ethanol. And that function and mechanism of mitophagy in yeast remain unclear. In the current study, the effect of ethanol on the expression of *ATG32* and *ATG11* genes was firstly inquired. Then the differences in the cell viability, cell cycle and ROS content between wild type and *atg32* and *atg11* mutants were assessed. The type and source of ROS in yeast were also further inquired in the study.

## Materials and Methods

### Strain and Maintenance Medium

The yeast strain used in this study was BY4742 which was supplied by Pro. Zhiwei Huang of East China University. The mutants of *atg11* and *atg32* were purchased from the company (Invitrogen, USA). The mutant *atg11* was constructed by hygromycin stripe homologous replacement the gene of *ATG11* and the same was the mutant *atg 32*. Then the cells of mutants were grown on yeast peptone dextrose (YPD) agar medium with 200 μg/ml geneticin G418. YPD agar medium contained 2% glucose, 1% peptone, 5% yeast extract and 2% agar. Yeast cells of wild type could not grow on YPD medium contained G418 whereas cells of mutants could survival from the medium with G418. Fresh cells grown on YPD medium slants for 24 h were used in all experiments.

### Fermentation Conditions

The yeast cells for all experiments, as starter cultures, were grown for 12 h in 100 ml of YPD liquid medium at 30°C with 180 rpm. The yeast cells, as the experimental cultures, were inoculated with 5 × 10^5^ CFU ml^-1^ of starter culture solution. According to the methodology [30], the fermentation of cells was grown in YPD liquid medium at 30°C with 180 rpm. 10% ethanol (v/v) by adding absolute ethyl alcohol was used as the ethanol stress. 100 μM 2-methoxyestradiol (2-ME) and 3-amino-1,2,4-triazole (3-AT), 5 μM Rapamycin (Rapa), 10 μM methyladenine (3-MA), 2.5 mM glutathione (GSH), 1 mM N-acetyl-cysteine (NAC), 50 μM antimycin A (AntiA) and rotenone (Rote) were applied to treat yeast cells, respectively. The yeast cells as samples were collected at 1, 2, and 24 h for assessing fermentation and growth parameters. The alcohol content in fermentation medium was measured by method of densitometer.

### Assessment of Mortality and Plasma Membrane Integrity

The mortality of cell and the plasma membrane integrity were both assessed by the fluorescent dye propidium iodide (PI) (Molecular Probes, Sigma, USA) vital staining with minor adaptations [31, 32]. PI is a nuclear staining reagent and it is an analog of ethidium bromide that releases red fluorescence upon insertion of double-stranded DNA. PI can not pass through the cell membrane of living cells whereas can traverse the broken membrane of the death cell. Briefly, yeast cells (10^6^ cells ml^-1^) were stained by 20 μM PI for 10 min at 37°C. The mortality of cells was measured by flow cytometry. The plasma membrane integrity of cells was observed by fluorescence microscope. The fluorescence intensity were measured by fluorescence microplate with 540 nm excitation wavelength and 590 nm emission wavelength.

### Assessment of Mitochondrial Membrane Potential

Based on described elsewhere [33], the mitochondrial membrane potential of cells was detected by rhodamine 123 (Rh123). Rh123, as a cationic dye, can transfer into the mitochondrial matrix with help of mitochondrial membrane potential. So, the fluorescence of the yeast cells was weak under normal circumstances. On the contrary, fluorescence of Rh123 in broken yeast was high because Rh123 was released from mitochondria by disruption of mitochondrial membrane potential.

### Cell Cycle Analysis

 According to a previously described procedure, cell cycle analysis was also performed by PI [31]. The yeast cells were harvested, washed, and fixed with ethanol (70%, vol/vol) for 30 min at 4°C. Then, the cells were destructed cell wall by sonication and followed by treatment with RNase for 2h at 37°C in 50 mM sodium citrate buffer (pH 7.5). Subsequently, the cells were incubated with proteinase K (1 mg per 10^6^ cells). Cell DNA was then stained overnight with PI (Molecular Probes, Sigma) at 4°C. Flow cytometry was used to determined cells in each phase of the cell cycle.

### Assesement of Intercellular Reactive Oxygen Species

H_2_O_2_ was monitored by 2,7-dichlorodihydrofluorescein diacetate (DCFH-DA) (Molecular Probes, Sigma) essentially as described elsewhere [34]. DCFH-DA does not fluoresce and can traverse the cell membrane freely. DCFH-DA can be hydrolyzed by esterase to 2, 7-dichlorofluorescin (DCFH) which is arrested in an actively respiring cell. DCFH was oxidized by H_2_O_2_ to a fluorescent compound DCF. Dihydroethidium (DHE) (Molecular Probes, Sigma) as a probe was used to detect O_2_^•-^. The yeast cells (10^6^ cells ml^-1^) were stained by 10 μM DCFH-DA or 4 μM DHE for 10 min at 30°C in dark. The fluorescences of DCF and DHE were detected by fluorescence microscope and flow cytometry.

### Flow Cytometry Analysis

Calibur flow cytometer (Becton, Dickinson and Company, USA) was used to detected fluorescence-activated cell sorter (FACS). The flow cytometer equipped with an argon ion laser emitting a 488 nm beam at 15 mW. The green fluorescence was detected by a 550 nm/long-pass dichroic mirror with a 525 nm/band-pass filter. The red fluorescence was monitored by a 590 nm/long-pass with a 620 nm/band-pass filter. The green and red fluorescences were both collected by means of a 488 nm blocking filter. The green fluorescence (FL1 log) and the red fluorescence (FL2 log) were defined to measure by an acquisition protocol on a 4-decade logarithmic scale. The software for the Flow Jo System II acquisition and software cell quest pro were used to analyze data (20,000 cells per sample).

### qRT-PCR

The RNA was extracted from the yeast cells of wild type by means of Trizol kit (Invitrogen). The destruction of cells was used by heat shock treatment with 15 min at 42°C followed by 3 min at 95°C. Then, the SuperScript III Platinum Two-Step qRT-PCR kit with SYBR green (Invitrogen) was used to reverse transcribed the total RNA. The reverse-transcribed RNA was used as a template to amplify the genes, using primers to the *ATG11* gene (sense, 5-TACAATCGTCTCCCTCGGTG-3; antisense, 5-TCCAAAGTGACAATTCTGCCTA-3), the *ATG32* gene (sense, 5-AAATGTCGTTTCACCGTCTCA-3; antisense, 5-ACCATCATCATCTTGCTCGTTA-3) and to the *ACT1* gene (sense, 5-GGATTCTGAGGTTGCTGCTTT-3; antisense, 5-TGACCCATACCGACCATGATAC-3). Real-time quantitative transcription polymerase chain reaction (qRT-PCR) was analyzed the expression of the expression of *ATG11* and *ATG32* genes by ABI Stepone plus (ABI). These results were normalized to the reference gene *ACT1*. Livak method or the 2^-ΔΔCT^ method was used to analyze the data [35]. The equation was calculated as follows: ΔΔCT = (C_T_(target gene) − C_T_(reference gene)) test − (C_T_(target gene) − C_T_(reference gene)) calibrator. CT was referred to threshold cycle.

### Statistical Analysis

Data were presented as means ± standard deviations (SD) and the means were from at least three independent assays. To analysis the differential significance of the data, Student’s t test were carried out by Statistical Product and Service Solutions 19.0 (SPSS). Statistically significant or very significant were represented by *p* values of less than 0.05 or 0.01, respectively and were added a single or double asterisk at the tops of the columns in the figures.

## Results and Discussion

### Ethanol Stress Induces Mitophagy

**Fig. 1:**
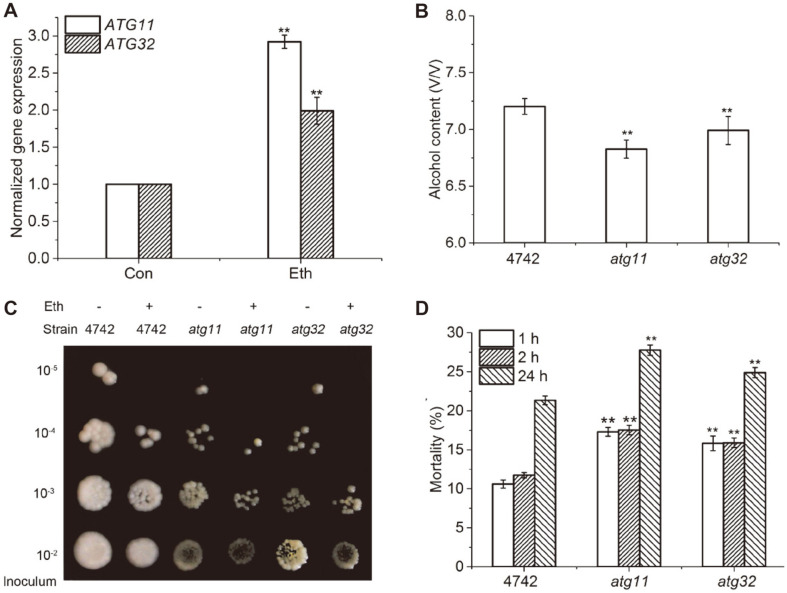
Ethanol stress increased expression of the *ATG11* and *ATG32* genes and fermentation profiles. Normalized fold expression of the *ATG11* and *ATG32* were evaluated by qRT-PCR in *S. cerevisiae* under ethanol treated for 4 h (**A**). The gene of actin (*ACT1*) was used as internal reference. Ethanol yield of S.cerevisiae grown on YPD medium for 3 d at 30°C were measured by density bottle method (**B**). Colony morphology of yeasts grew on YPD medium with or without 10% ethanol for 3–5 d at 30°C (**C**). Mortality of cells under mid-log phase was treated with 10% ethanol for 1, 2, and 24 h. Then the yeast were stained with PI and analyzed by flow cytometry (**D**). Values indicated mean ± standard deviation (*n* = 3). Statistical significance was determined by a Student’s t test. Double star represented very significant between the wild type and mutants (*p* < 0.01).

It is established that ethanol accumulates gradually in *S. cerevisiae* during the fermentation process [24]. In order to confirm whether mitophagy is induced by ethanol stress, the expression of *ATG11* and *ATG32* genes under ethanol stress was measured by qRT-PCR. Results indicated that the expression of *ATG11* and *ATG32* genes was substantially increased by ethanol stress (*p* < 0.01) (Fig. 1A). The expression of *ATG11* and *ATG32* in yeast cells was increased to 278.3 % and 194.0% by ethanol stress, respectively. Therefore, ethanol stress induces mitophagy and other selective forms of autophagy.

*S. cerevisiae* is a traditional fermentation microbe and has fewer mitochondria used for degradation compared to other yeast cells. It is difficult to induce mitophagy in a fermentable medium. Therefore, mitophagy is induced when cells are exposed to rapamycin (the Tor inhibitor) or nitrogen deficiency after pre-culturing in a fermentation medium [10]. In the current study, ethanol increased the expression of *ATG11* and *ATG32* genes within 4 h (Fig. 1). Moreover, it is well known that *ATG11*, as a scaffold molecule, particpates in many forms of selective autophagy [19-23]. Therefore, there is evidence that ethanol induces mitophagy as well as other forms selective autophagy.

### Mitophagy Increases Alcohol Output and Decreases Mortality in Yeasts Exposed to Ethanol Stress

Although *ATG11* contribute to many selective autophagy [20-23], the mutant *atg11* is known to inhibit mitophagy [36]. In order to investigate the function of mitophagy during ethanol stress, the alcohol production of wild type and mitophagy mutants *atg11* and *atg32* was assessed in normal YPD medium. Results indicated that although the growth curves of the wild type and mutants were not different in the absence of ethanol stress (Fig. S1) the alcohol output of *atg11* and *atg32* was significantly lower than that of the wild type (Fig. 1B). In contrast, the colony sizes of all strains were smaller under ethanol stress (Fig. 1C), and the colony sizes of the mutants were much smaller compared to wild type colonies (Fig. 1C). In addition, ethanol stress increased the cell death in all strains (Fig. 1D) and the mortality of *atg11* and *atg32* was remarkably higher than that of wild type under ethanol stress (Fig. 1D). For example, the mortality of wild type, *atg11* and *atg 32* with ethanol for 1h was 10.6%, 17.8%, and 15.8%, respectively. These results suggest that mitophagy increases alcohol production by protecting yeast injure from ethanol stress.

The accumulation of ethanol in a medium inhibits the growth and viability of yeast cells [30,37,38]. And a similar finding was made in the present study. Ethanol decreased the growth rate and promoted cell death in all strains (Figs. 1C and 1D). In addition, it has been reported that the biomass yield of the *atg32* is decreased, whereas the output of ethanol is increased [28]. Similarly, *atg32* and *atg11* were decreased the growth rate and were increased the death rate by ethanol stress (Figs. 1C and 1D). In contrast, alcohol output of mitophagy mutants was decreased (Fig. 1B). It is suggested that the death of yeasts by ethanol stress occurs during the early fermentation stage. Alcohol production requires the living yeast to transfer carbohydrate to alcohol throughout the entire fermentation process. For example, reducing agents increased the alcohol output (Fig. S4) through an attenuation of the cell death (Fig. S2A). In contrast, antioxidase inhibitors reduced alcohol yield (Fig. S4) by increasing the cell death (Fig. S2B).

**Fig. 2:**
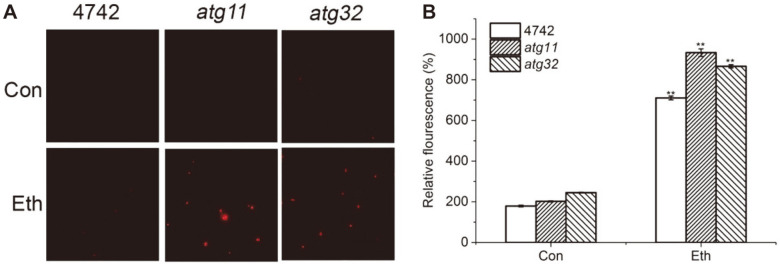
Ethanol stress injures membrane integrity of yeast. Membrane integrity of WT strans, *atg11* and *atg32* mutants with 10% ethanol for 2 h were stained by PI, and then analyzed by fluorescence microscope (**A**) and fluorescence microplate reader (**B**). Values indicated mean ± standard deviation (*n* = 6). Statistical significance was determined by a Student’s t test. Double star represented very significant between the wild type and mutants (*p* < 0.01).

Mitophagy Contributes to the Maintenance of Mitochondrial Membrane Potential and Cell Cycle of Yeast Cells Ethanol gradually accumulates in the culture broth during the process of fermentation. *S. cerevisiae*, an efficient ethanol producer, has a high tolerance to ethanol [38-40]. However, high levels of ethanol eventually disrupts cell membrane integrity [37-39]. As seen in Fig. 2A, ethanol stress increased fluorescence intensity in all strains. However, the relative fluorescence intensity of *atg11*, *atg32* was greater compared to the wild type. Compared to wild type, the relative fluorescence intensity of *atg11*, *atg32* was 131.4% and 123.2% under ethanol stress, respectively (Fig. 2B). So, ethanol stress destroys membrane integrity in all strains and mitophagy is involved in the maintenance of yeast membrane integrity.

**Fig. 3:**
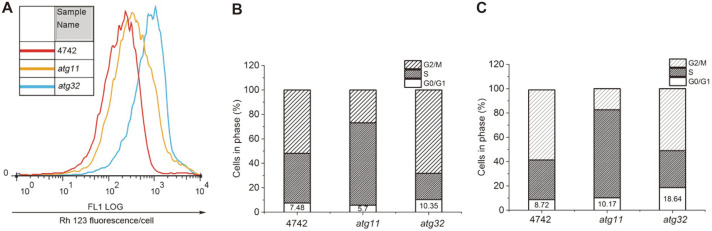
Effects of mitophagy on mitochondrial membrane potential and cell cycle of *S. cerevisiae* under ethanol stress. Yeasts were stained with Rh123 to detect mitochondrial membrane potential. The fluorescence of *S. cerevisiae* with ethanol for 4h were obtained by FACS green fluorescence histograms (FL1 log) (**A**). Cell cycle profile of the WT, *atg11* and *atg32* grown on YPD without (**B**) or with (**C**) 10% ethanol for 4h were analyzed by flow cytometry analysis. The percentages of wild type and mutants in G1/G0, S and G2/M phases were summarized from flow cytometry analysis, respectively.

The mitochondrial inner membrane exhibits vital roles in mitochondrial function, such as with the electron transfer chain and transmembrane transport [41]. To further explore the impact of ethanol-induced mitophagy, mitochondrial membrane potential of *S. cerevisiae* were analyzed. Compared with wild type, the mitochondrial inner membrane of the mitophagy mutants especially *atg32*, exhibited greater damage from ethanol stress (Fig. 3A). This indicates that mitophagy assists in maintaining the integrity of the mitochondrial inner membrane. Therefore, ethanol stress not only destroys the plasma membrane but also damages the inner membrane of mitochondrial.

It has been reported that nitrogen deficiency arrests the cell cycle in G1/G0 and that autophgy is required for G1/G0 quiescence [30, 42]. In comparison to the control fermentation, ethanol stress caused cell cycle arrest in phases G0/G1 in all strains (Figs. 3B and 3C). The cell cycle arrest of mutants was more severe compared to wild type (Figs. 3B and 3C). Therefore, mitophagy contributes to ethanol tolerance in yeast by accelerating cell division, maintaining integrity of cell membrane and mitochondrial inner membrane [37, 43].

### Ethanol Stress Induces Production of H_2_O_2_ and O_2_^•-^ in *S. cerevisiae*

**Fig. 4:**
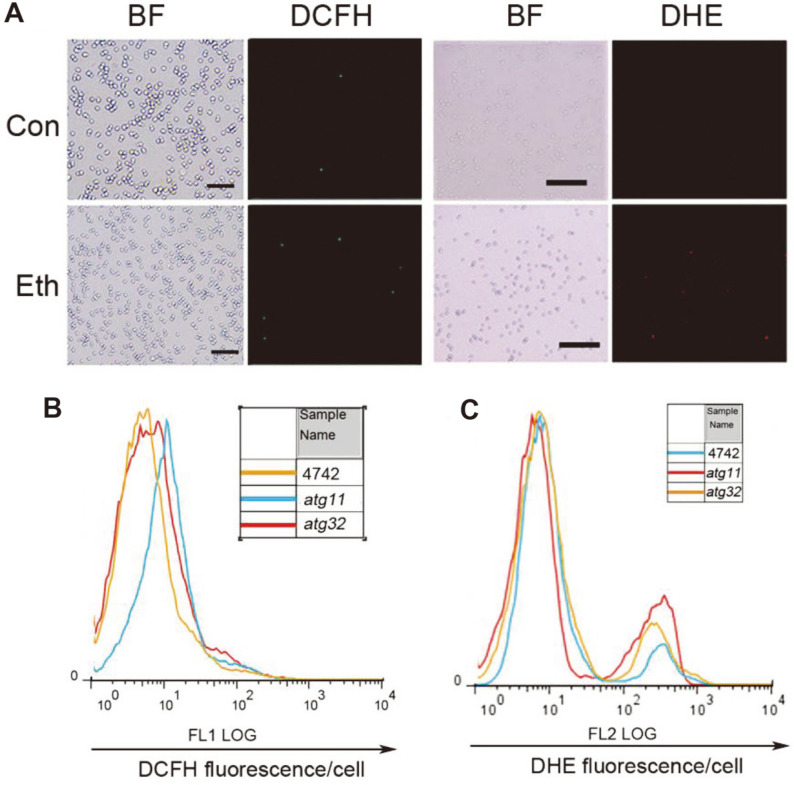
Production of H_2_O_2_ and O_2_^•-^ was changed in strains under ethanol stress. Production of H_2_O_2_ and O_2_^•-^ in wild type with ethanol for 2 h was stained by DCFH for 30 min or DHE 10 min, respectively (**A**). Production of H_2_O_2_ (**B**) and O_2_^•-^ (**C**) in WT, *atg11* and *atg32* with 10% ethanol for 24 h was stained by DCFH or DHE and analyzed by flow cytometry. Overlay of fluorescence-activated cell sorter green or red fluorescence histograms (FL1 log) was obtained.

It is established that ethanol activates oxidative stress in yeast cells during fermentation [5, 24, 38]. To evaluate whether ROS accumulation was associated with ethanol stress, ROS production was measured by fluorescence staining and flow cytometry. Production of H_2_O_2_ and O_2_^•-^ was induced by ethanol stress in wild type (Fig. 4A). H_2_O_2_ production in *atg11*, *atg32* was higher than that in wild type. The production of H_2_O_2_ in *atg32* during ethanol stress was greatest (Fig. 4B). Similarly, the O_2_^•-^ content in mutants was also higher than that in wild type (Fig. 4C). However, the difference in O_2_^•-^ between wild type and mutants was not as obvious as that seen with H_2_O_2_. Therefore, ethanol stress contributes to the accumulation of ROS and mitophagy mutants accumulate greater levels of ROS.

### ROS Regulates Mitophagy during Ethanol Stress

**Fig. 5:**
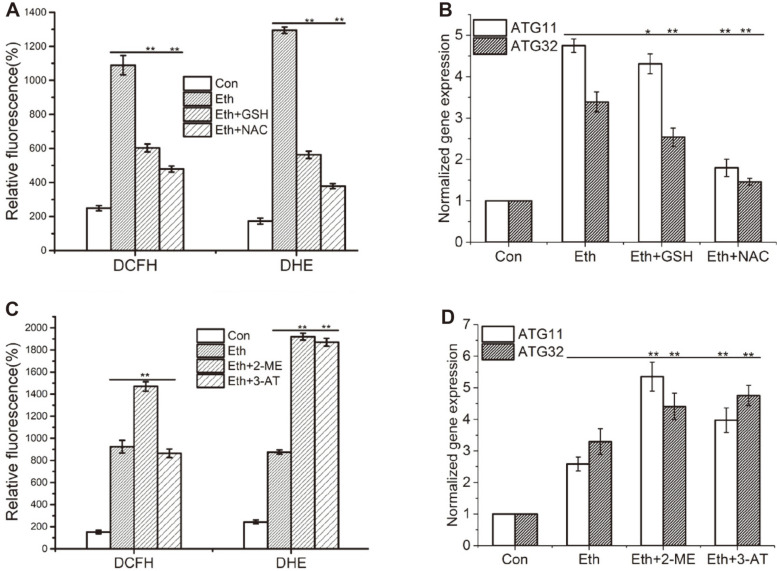
Reductants and antioxidase inhibitors regulated expressions of the *ATG11* and *ATG32* genes and contents of H_2_O_2_ and O2•- in *S. cerevisiae* cells under ethanol stress. The level of H_2_O_2_ and O_2_^•-^ in wild type cells treated the reductants (**A**) or antioxidase inhibitors (**C**) for 2h were stained by DCFH or DHE, respectively. The relative fluorescence was summerized in A and C (*n* = 6). The reductants further decreased expressions of *ATG11* and *ATG32* (**B**) and the antioxidase inhibitors induced expression of *ATG11* and *ATG32* (**D**). Normalized fold expression levels of the *ATG11* and *ATG32* genes were evaluated by qRT-PCR in *S. cerevisiae* with ethanol treated for 4h. The gene of actin, *ACT1*, was used as internal reference. Values indicated mean ± standard deviation (*n* = 3). Statistical significance was determined by a Student’s t test. Double star or single star represented very significant (*p* < 0.01) or significant (*p* < 0.05).

Moderate level of ROS, as signal molecule, regulates autophagy [30]. In order to clarify the complex relationship between ROS and mitophagy under ethanol stress, reducing agents and inhibitors of antioxidase were used to alter ROS content.The common reducing agents GSH and NAC clearly reduced the production of H_2_O_2_ and O_2_^•-^ when applied to ethanol treated yeast cells for 2 h (Fig. 5A). Corresponding with a decrease of ROS, GSH and NAC significantly decreased gene expression of *ATG11* and *ATG32* (Fig. 5B). For example, GSH decreased the expression of *ATG11* and *ATG32* to 91.5% and 75.8% during ethanol stress, and NAC decreased *ATG11* and *ATG32* expression to 38.3% and 44.1%. Therefore, GSH and NAC decrease mitophagy by attenuating the production of H_2_O_2_ and O_2_^•-^.

2-ME, as the inhibitor of Mn-SOD in mitochondria, resulted in an increase of O_2_^•-^ in mitochondria (Fig. 5C). 3-AT, as the inhibitor of CAT, contributed to the rise of H_2_O_2_ in cytoplasm (Fig. 5C). Results indicated that 2-ME and 3-AT both increased expression of *ATG11* and *ATG32* genes exposed to ethanol stress (Fig. 5D). The expression of *ATG11* and *ATG32* increased to 207.4 % and 134.1 % by 2-ME. And 3-AT increased *ATG11* and *ATG32* expression to 153.9% and 144.7 %. So, 2-ME and 3-AT increase mitophagy by augmenting the production of H_2_O_2_ and O_2_^•-^.

H_2_O_2_ in the yeast mitochondrial matrix plays a signaling role in ethanol tolerance through the activation of Yap1p [5]. It is well known that ROS regulates mitophagy. For example, mitophagy is positively affected by cellular oxidative stress [10]. NAC and cysteine inhibited mitophagy due to increasing cellular levels of reduced glutathione [44] and also decreased the expression of Atg32 [45]. The current results support these findings. Both GSH and NAC inhibited the expression of the *ATG32* and *ATG11* genes (Fig. 5B) by reducing the production of H_2_O_2_ and O_2_^•-^ (Fig. 5A). In contrast, the antioxidase inhibitors 2-ME and 3-AT increased expression of the *ATG32* and *ATG11* genes (Fig. 5D) due to increase production of H_2_O_2_ and O_2_^•-^ (Fig. 5C). Therefore, levels of H_2_O_2_ and O_2_^•-^are capable of regulating mitophagy in yeast exposed to ethanol stress.

**Fig. 6:**
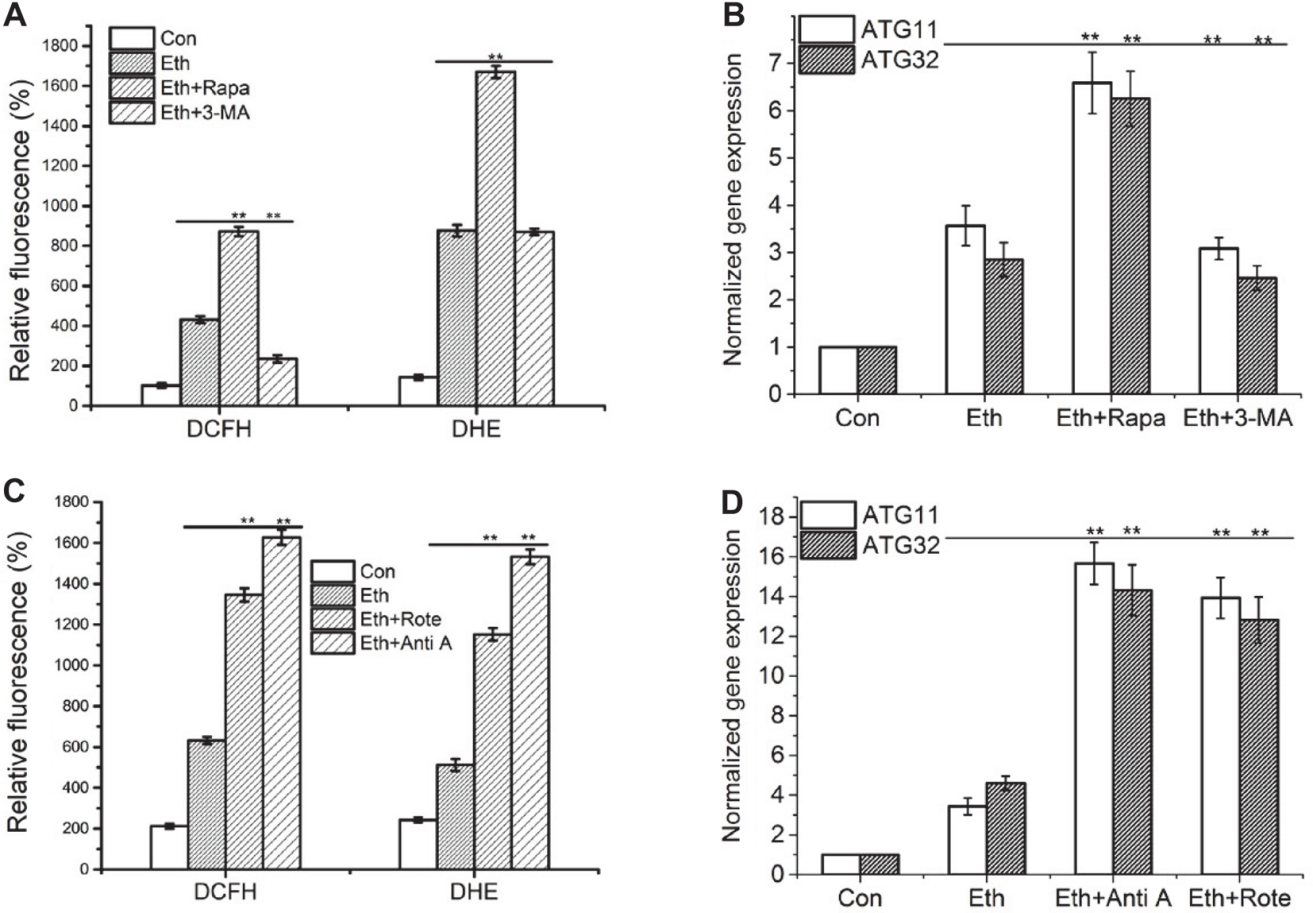
The respiratory chain inhibitors or autophagy regulators regulated expression of the *ATG11* and *ATG32* genes and contents of H_2_O_2_ and O2•- in *S. cerevisiae* cells under ethanol stress. Contents of H_2_O_2_ and O_2_^•-^ in wild type cells treated with Rapa and 3-MA (**A**) or Rote and Anti A (**C**) for 2 h were stained by DCFH or DHE, respectively. The relative fluorescence was summarized in A and C (*n* = 6). Rapa and 3-MA had dual functions on expression *ATG11* and *ATG32* (**B**). Rote and Anti A further induced expression of *ATG11* and *ATG32* genes (**D**). Normalized fold expression levels of the *ATG11* and *ATG32* gene were evaluated by qRT-PCR in *S. cerevisiae* under ethanol treated for 4h. The gene of actin, *ACT1*, was used as internal reference. Values indicated mean ± standard deviation (*n* = 3). Statistical significance was determined by a Student’s t test. Double star represented very significant (*p* < 0.01).

Lastly, to demonstrate the ROS regulation of mitophagy in *S. cerevisiae* under ethanol stress, an inhibitor of autophagy 3-MA and inducer of autophagy Rapa were added to the medium under ethanol stress, respectively. Results indicated Rapa promoted the increase expression of *ATG11* and *ATG32* genes to 184.7% and 219.6% under ethanol treatment for 2 h, respectively (Fig. 6B). Therefore, the inducer of autophagy is also a mitophagy activator. Similarly, Rapa promoted the production of H_2_O_2_ and O2-. under ethanol treatment for 2 h (Fig. 6A). In contrast, an inhibitor of autophagy 3-MA decreased the expression of *ATG11* and *ATG32* genes under ethanol stress (Fig. 6B). The increases of H_2_O_2_ and O2-. level by ethanol were also decreased by 3-MA (Fig. 6A). In addition, Rapa increased the death rate of yeast cells whereas 3-MA decreased the rate (Fig. S3A). In summary, the inducing of mitophagy by ethanol activates yeast cell death, results in decrease of alcohol output. Therefore, regulators of autophagy also control levels of mitophagy through altering levels of ROS molecules in yeast. And mitophagy responds to varying levels of mitochondrial oxidative stress to control the quality and quantity of mitochondria [7].

### The Inducing ROSs by Ethanol Are Mainly Derived from mtETC

Anti A and Rote are the inhibitors of complex III and complex I of mtETC. The result indicated that Anti A and Rote both increased the production of H_2_O_2_ and O_2_^•-^ (Fig. 6C) and increased the expression of *ATG11* and *ATG32* genes exposed to ethanol stress (Fig. 6D). Compared with ethanol stress, Anti A induced expression levels of *ATG11* and *ATG32* to 456.3% and 310.9%, and Rote increased *ATG11* and *ATG32* expression to 387.8% and 278.3%. In addition, Anti A and Rote augmented yeast cell death (Fig. S3B) and substantially lowered the alcohol yield (Fig. S4). Therefore, ROSs derived from mtETC regulate mitophagy and play a critical role in mediating alcohol production.

Mitophagy facilitates H_2_O_2_ and O_2_^•-^ clearance during ethanol exposure. Similar conclusion has been reported by Kurihara *et al*. [13] where mitophagy decreases ROS production from mitochondria. In addition, several studies have reported that mitophagy promotes the elimination of ROS through the removal of damaged mitochondria [9, 10, 13, 47]. Elevated respiration rates stimulate mitophagy degradation of the complex I equivalent and complex III of mtETC [27]. Therefore, the complex I equivalent and complex III are key mediators of ethanol stress related changes. Anti A and Rote, as inhibitors of complex III and complex I of the mtETC, were used to increased ROS. Further investigation revealed that Anti A and Rote activated mitophagy (Fig. 6D) by means of increasing the production of ROS in mitochondria (Fig. 6C). It is concluded that ROSs derived from mtETC participate in the regulation of ethanol-induced mitophagy. Similar results of Anti A induced mitophagy have been reported [46].

In conclusion, mitophagy is a complex cellular activity in the yeast. Mitochondrial oxidative stress induced by mild ethanol, as a prosurvival response during the early stages of fermentation, activates mitophagy. Mitophagy contributes to the clearance of ROS and protects yeast cells from ROS damage. These phenomena results in improved cell membrane integrity, maintenance of the mitochondrial membrane potential, decreased cell mortality, and ultimately enhanced alcohol output. Mitophagy in yeasts is regulated by ROS level in the cell during ethanol exposure. In addition, recent research indicates that an *ATG32*-independent pathway also contributes to the removal of mitochondria [48]. Whether Atg32 independent mitophagy induced by ethanol during fermentation requires further study.

## Supplemental Material



Supplementary data for this paper are available on-line only at http://jmb.or.kr.
