# Supplementary material for: Mitophagy Improves Ethanol Tolerance in Yeast: Regulation by Mitochondrial Reactive Oxygen Species in Saccharomyces cerevisiae
Source: J Microbiol Biotechnol. 2020 Sep 25;30(12):1876–84. doi: 10.4014/jmb.2004.04073 (PMC9728279; doi:10.4014/jmb.2004.04073)
Supplement: Supplementary file 1 [file JMB-30-12-1876-supple.pdf]

J. Microbiol. Biotechnol.
https://doi.org/10.4014/jmb.2004.04073

Figure S1

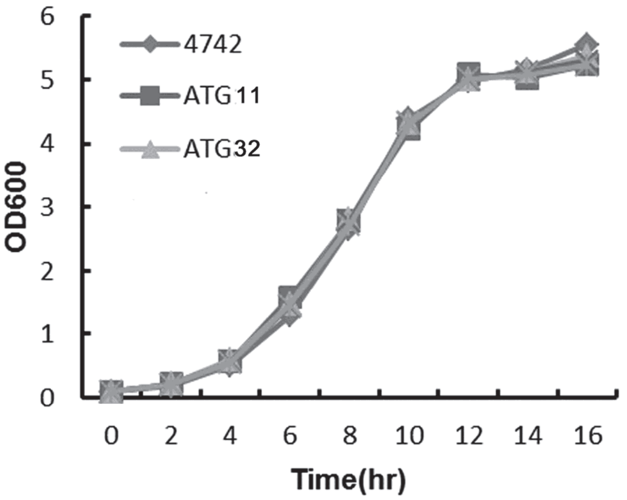


**Fig.S1 Growth curve of wild type and mitophagy mutants of *S. cerevisiae.*** All strains of BY4742 (*S. cerevisiae*) were grown on YPD medium at 30℃, 180 rpm for 16h. Optical density (OD) at 600 nm were measured every 2h.

Figure S2

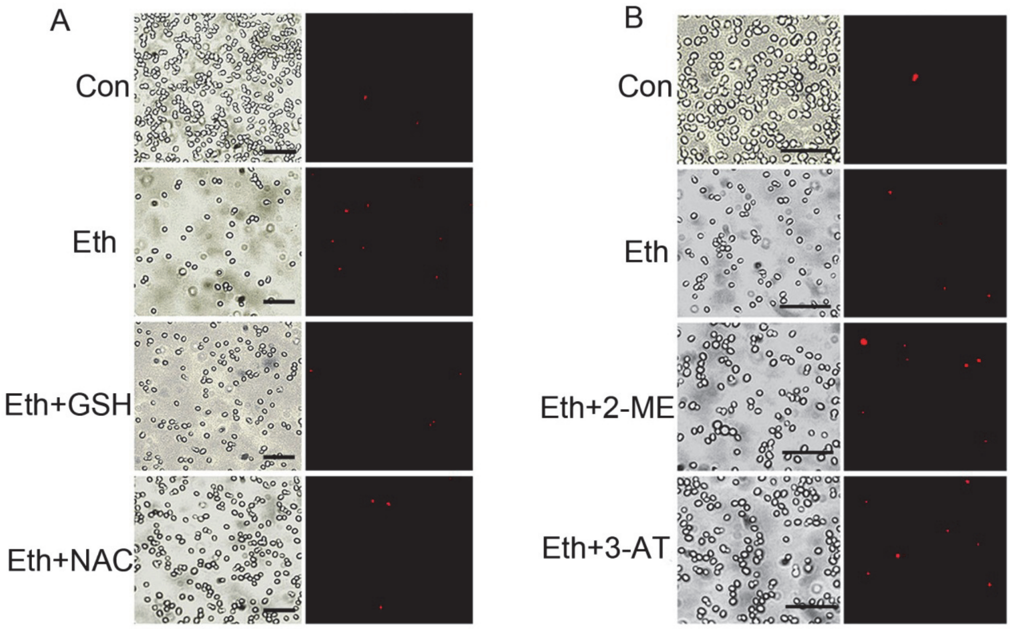


**Fig.S2 Effects of reductants and inhibitors of antioxidant enzyme on death of *S. cerevisiae* cells during fermentation**. GSH and NAC decreased the death of yeast under ethanol stress (A). 2-ME and 3-AT increased the death of yeast under ethanol stress. Wild type of yeasts in logarithmic phase were grown YPD medium with 2.5 mM GSH、1 mM NAC, 100 uM 2-ME and 100 uM 3-AT under 10% ethanol for 2 h, respectively. Yeasts above shown were stained by PI for 10 min and then observed by fluorescence microscope.

.

Figure S3

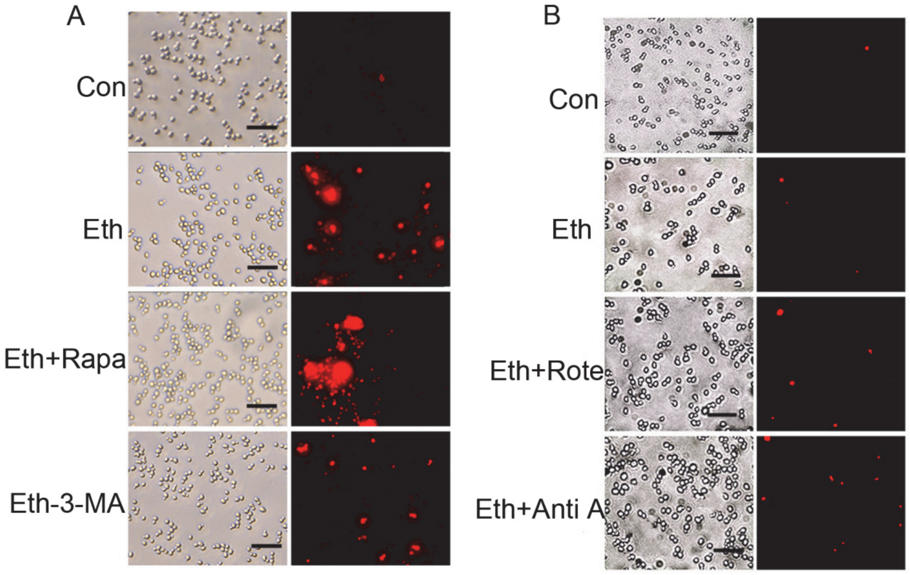


**Fig.S3 Effects of autophagy regulators and inhibitors of respiratory chain in mitochondria on death of *S. cerevisiae* cells during fermentation**. Rapa and 3-MA regulated the death of wild type of yeast cells under ethanol stress (A). Rote and anti A increased the death of yeast under ethanol stress (B). Wild type of yeasts in logarithmic phase were grown on YPD medium with 5 uM Rapa, 10 uM 3-MA, 50 uM Rote and Anti A under 10% ethanol for 2 h, respectively. Yeast cells above shown were stained by PI for 10 min and then observed by fluorescence microscope.

Figure S4

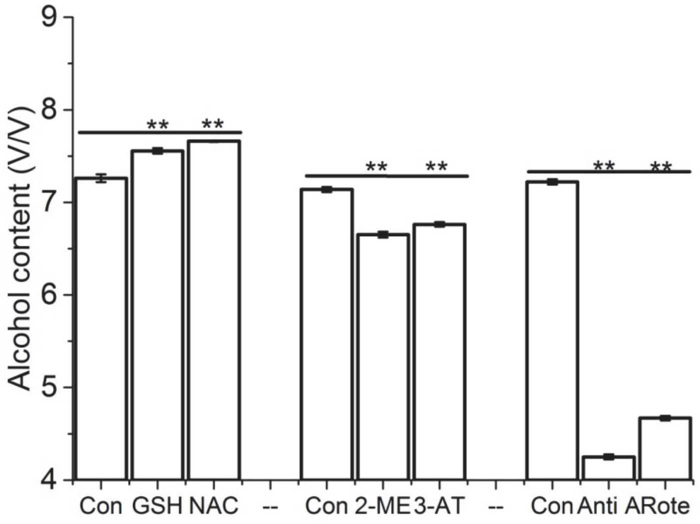


**Fig.S4 Change of ROS contents regulated the alcohol yield of yeasts**. Ethanol yield of wild type yeast cells grown on YPD medium for 3 d at 30℃ were measured by density bottle method. Values indicated mean ± standard deviation (n=3). Statistical significance (**, $P < 0.01$) was determined by a Student's t test.
